# A new method for the evaluation of makeup coverage using hyperspectral imaging

**DOI:** 10.3389/fchem.2024.1400796

**Published:** 2024-09-10

**Authors:** Carl Blaksley, Kumiko Udodaira, Alexandre Nicolas, Marco Casolino

**Affiliations:** ^1^ L’Oréal Research & Innovation, Kawasaki, Kanagawa, Japan; ^2^ RIKEN, Wako, Saitama, Japan; ^3^ Istituto Nazionale di Fisica Nucleare, Sezione di Roma Tor Vergata, Rome, Italy; ^4^ Dipartimento di Fisica, Universitá degli Studi di Roma Tor Vergata, Rome, Italy

**Keywords:** hyperspectral imaging, spectral imaging, spectral analysis, color evaluation, product evaluation

## Abstract

**Introduction:**

The *coverage* of a makeup foundation is a perceived attribute which is not captured by opacity or any other single optical property. As previous instrumental measurements do not allow us to consistently compare one product to another, we have begun exploring new parameters and analysis methods made available by hyperspectral imaging. Presumably, the coverage of makeup comes from the change in color, homogeneity, and evenness over the face after application, and the ability of the product to hide spots and other blemishes.

**Methods:**

As a starting point to unravelling this complex topic, we define a homogeneity factor $\alpha_{HF}$ which measures the change in the homogeneity of the spectra using the distribution of spectral angles in the face. We likewise define a spectral shift factor $\beta_{SF}$ which indicates the degree of spectral change after product application. To test these new parameters and the overall analysis method, we applied them to the HSI validation dataset which contains data for three makeup foundation products of different coverage levels applied to 9 models.

**Results:**

We find that $\alpha_{HF}$ correlates with the sensory ranking of coverage. Similarly, the parameter $\beta_{SF}$ correlates with the visible color change induced by the product, and we can map the three products into distinct categories based on their effect on $\alpha_{HF}$ and $\beta_{SF}$ .

**Discussion:**

Nevertheless, the homogeneity factor $\alpha_{HF}$ does not fully describe coverage, and in the variability in the product effect from model to model we find evidence that we must also account for the relative color difference between the model’s skin tone and the product shade among other factors.

## 1 Introduction

The *coverage* of a makeup foundation is a perceived attribute which goes beyond opacity or any other single optical property, and, as with many such attributes in the cosmetics field, the appreciation of a given coverage effect varies with age, culture, and trends. To date, makeup coverage is generally ranked by expert sensory evaluation, which works well when product differences are large and it is sufficient to test only one or two products on a small number of models. The goal in connecting coverage to a set of instrumental measurements is to provide a repeatable and reliable evaluation which we can apply systematically to a number of products throughout large studies. To do this, we must first address the question, “What is coverage?”.

Focusing on the visual aspects only, we expect that the perceived coverage of a product such as makeup foundation relates to the color change caused by the product, the change in color homogeneity and evenness over the face after application, and the ability of the product to hide spots and other blemishes. While it is likely distinct from the overall coverage effect, the color change itself is important with regard to the perception of the product effect being more or less desirable. For example, some trends or consumer segments emphasize a “natural” appearance with little visible skin color change, but at the same time these users might want a good blemish-hiding effect. On the other hand, other trends might emphasize a more “made-up” appearance as being desirable.

Separated from the color change, the color homogeneity is likely to be the most closely linked to a simple notion of coverage. When we speak of homogeneity it is important to keep in mind two distinct properties, the homogeneity of the color space, that is the number of distinct colors in a region, and the evenness of the spatial distribution of colors in the area, that is the texture or color evenness. If we conceive of coverage as an attribute which is about the hiding of blemishes, spots, pores, or wrinkles and the smoothing out of the skin tone, then understanding the product effect on the color and spatial homogeneity are the first entry points to decoding it.

In addition, the degree of perceived coverage effect will likely also depend on the initial skin condition. What we mean by this is that if coverage is an *improvement* in condition rather than the obtaining of a certain state, the *perceived* coverage is in a sense the degree of difference between the bare skin and the made-up effect. Naturally then, the perceived coverage effect will depend on how much there is to cover in the first place, and so will depend on the user and the matching of the product to the user, as much as on the product itself.

With all of this in mind, we nonetheless would like to find some instrumental measurement(s) which we can use to decode the coverage ability of makeup products. Fundamentally, this analysis method must correlate with the sensory evaluation results, and also be sensitive enough to distinguish small differences in product action with a reasonable sample size. The previous generation of methods for coverage analysis relied on parameters such as the volume of the $L^{*}a^{*}b^{*}$ distribution in a defined Region Of Interest (ROI), $\sigma_{L^{*}}\sigma_{a^{*}}\sigma_{b^{*}}$ , which we refer to as the Coxello Index. The advantage of this measurement is that it is easily available from existing color images, and it intuitively relates to the color homogeneity within the region. In practice, however, the change in Coxello Index after product application is not strongly correlated to the sensory evaluation of coverage and we are not able to consistently separate product effects with this parameter.

There are other attempts to develop a color-based parameter for coverage evaluation in the literature. For example, in (Batres et al., 2019) they compared the results of an image scoring test for “skin evenness” with two different measures of homogeneity, the Haralick homogeneity (Haralick et al., 1973) based on the distribution of L values and their own parameter, similar to the variance of the $L^{*}a^{*}b^{*}$ values added in quadrature, over ROI covering the cheek and forehead. They did not find a correlation between the skin evenness scoring and Haralick homogeneity, and they found that their $L^{*}a^{*}b^{*}$ -based homogeneity correlated with the skin evenness, but only on the cheek (not on the forehead). Their newly introduced parameter is similar to the Coxello Index, which we know can distinguish between before and after product application, but not consistently between the effect of different products, and their results are consistent with our own experience of evaluating coverage using $L^{*}a^{*}b^{*}$ measurements.

The use of imaging spectrometers, sometimes referred to as hyperspectral imaging, opens up novel possibilities for the evaluation of cosmetic products (see, for example, (Zonios et al., 2001; Nishidate et al., 2004)). First, as a hyperspectral imager measures the reflectance spectrum within each pixel in the field of view, it allows us to analyze optical properties without the loss of information inherent in color imaging, or the bias of working under an arbitrary illumination. At the same time, having the pixel-by-pixel spectra allows us to also use the spatial distribution of the spectra in our analysis, which distinctly separates the analyses possible with a hyperspectral camera from what was capable with non-imaging spectrometers. Perhaps more importantly, the difficulty of working with large volumes of spectral data forces us to create a comprehensive data analysis system. With such a system in place, we have a framework in which to implement analysis methods that are up to the task of decoding complex attributes like coverage.

In this paper, we will discuss a new method for evaluating makeup foundation coverage based on the analysis of the distribution of spectral differences within a region. We will first discuss the basis of this method and demonstrate its use in some example cases. We then test the evaluation power of the developed method on data from a study in which we applied a set of 3 different makeup foundations, with different degrees of coverage, to a panel of 9 models. As each of the products in that test where also evaluated by sensory experts in terms of coverage, we can directly compare the results of our instrumental method to those from sensory evaluation.

## 2 Materials and methods

Our standard setup for *in-vivo* skin color measurement is the *Chromasphere* system (De Rigal et al., 2010; De Rigal et al., 2007; Huixia et al., 2012; Flament et al., 2017). The Chromasphere itself is an 80 cm diameter integrating sphere combined with a fiber-coupled light source, providing a diffuse illumination for color photography. In the past configuration, the Chromasphere mounts three 3CCD color cameras capturing evaluation images from a front, left-side, and right-side view of the face.

As an update to the capabilities of the Chromasphere system, we have designed and constructed a custom imaging spectrometer suitable for full-face *in-vivo* photography. This Hyperspectral Imager (HSI) replaces one or more of the 3CCD cameras in the standard Chromasphere setup and is a spectrally scanning instrument based around a Liquid Crystal Tunable Filter (LCTF). Functionally, the HSI combines the spectrum-measurement capabilities of a spectroradiometer with the spatial resolution of the previous 3CCD color camera. It therefore provides a color measurement which is independent of the illumination, while its spatial resolution allows for *post hoc* selection of ROI as well as for analyses of the spatial variance of the reflectance spectrum. We previously reported the complete design and performance evaluation of this prototype (Blaksley et al., 2021), and here we will give only a brief overview of the instrument.

The tunable filter used in our prototype instrument provides three selectable bandwidth ranges of 32 nm, 18 nm, and 10 nm Full Width at Half Maximum (FWHM) (measured at λ=555 nm) with a continuously variable central wavelength from 420 to 730 nm at a tuning accuracy of ± FWHM /10 nm. The spectral range of the HSI is from 420 nm to 730 nm with a measurement step of 10 nm. In this configuration, instrument acquires 32 spectral bands for one hyperspectral image in less than 9.8 s. The 32 ${cm}^{2}$ field of view maps onto a 2016 by 2016 pixel (4.1 Megapixels) monochromatic CCD camera, giving a spatial resolution on the order of 160 μ m, and the Signal-to-Noise Ratio (SNR) is better than 47 across the majority of the working spectral range.

**FIGURE 1:**
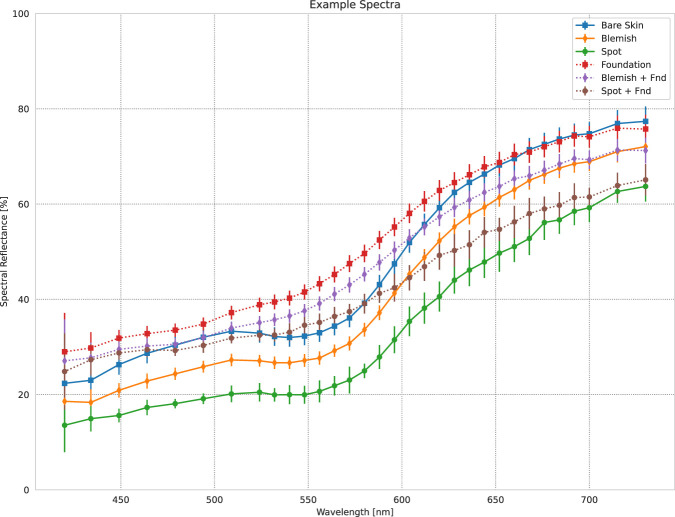
An selection of measured spectra from the data example presented in Figure 3. “Bare Skin” is the average spectrum from an area of clear skin on the model’s left cheek. “Spot” and “Blemish” are the average spectra from the dark spot and melasma on the model’s right cheek. “Foundation” is the “Bare Skin” area after application of makeup foundation $\left(T_{\text{imm}}\right)$ . Similarly, “Spot + Fnd” and “Blemish + Fnd” show the spectra of the respective regions after foundation application. The “Bare Skin” and “Foundation” spectra provide the reference spectra for further spectral angle comparisons, depending on the analysis being performed. Error bars indicate the standard deviation of the spectra across their respective ROI. Markers indicate actual measured wavelengths.

We developed a custom data acquisition software in C++ (ISO, 2003) and QT (QT, 2020) to operate this instrument and capture hyperspectral images, or *datacubes*, which encode the spatial and spectral information from the field of view into 3-dimensional voxels (two spatial and one spectral dimension). In Figure 1, we present a selection of spectra measured with this instrument, taken from the dataset of this study. The HSI exists as part of a complete data ecosystem combining the instrument with a comprehensive data analysis tool chain, and we do all data treatment and analysis using our own hyperspectral image analysis framework written in Python 3 (Python, 2020).

### 2.1 Dataset

The data which we will use in this study comes from the HSI validation test carried out in March 2019. At that time, we conducted a study consisting of 3 foundation formulas applied to a group of 9 models as an end-to-end test of the HSI system. As products, we selected 3 makeup foundation products each with a different level of coverage as evaluated by expert sensory evaluation, and for the models we selected 9 models with a higher density of spots from our regular panel of models. We did not attempt to select the models based on skin tone.

For each product, we captured a hyperspectral image of each model before $\left(T_{0}\right)$ and immediately after $\left(T_{\text{imm}}\right)$ product application according to the test protocol which we show in Table 1. In order to test the repeatability of the instrumental results, we studied each product twice, on different days. In addition, at each repetition two different operators took the same data in order to assess the reproducibility of the results. We organized these twelve tests (3 formulas × 2 repetitions × 2 operators) on 3 days over the course of 3 weeks. The end result of this study is a dataset of 216 hyperspectral images including both bare and made-up skin conditions. We previously presented an analysis of the repeatability and reproducibility results from this test, including a cross-comparison against measurements taken with other instruments in (Blaksley et al., 2022).

**TABLE 1:** Test protocol used in the HSI validation study.

1	Wash with cleansing oil and foaming cleanser (no moisturizer)
2	Moisturize with cosmetic water and milky lotion
3	Wait for 15 min
4	Perform $T_{0}$ measurement
5	Product application by operator
6	Wait 10 min for product to dry
7	Perform $T_{\text{imm}}$ measurement

### 2.2 A new method for analyzing coverage

The HSI validation dataset contains a large amount of data in which we applied makeup foundation products with known coverage levels, and so it provides the perfect stage on which to develop new methods for the analysis of makeup coverage. As discussed in the introduction, we propose a new method for evaluating makeup foundation coverage based on the analysis of the distribution of spectral differences within the face before and after the product application. We show real examples of the spectra of various skin features before and after application of a makeup foundation in Figure 1. When we speak of the difference between the spectra of such features, the first parameter which we can look to is the *spectral angle*, θ . Here the “angle” is that between the two spectra treated as n-dimension vectors:

$$\theta =\cos^{-1}\left(\frac{S_{a}\cdot S_{b}}{\left|S_{a}\|S_{b}\right|}\right)$$ (1)

where $S_{a}$ is some *reference spectrum* and $S_{b}$ is the spectrum under test, $S_{a}\cdot S_{b}$ is the dot product of the two vectors, and |S| is the vector length. The angle θ functions as a measure of the *difference* between the two spectra (here in units of degrees).

In this respect θ is similar to the color difference metric ΔE , but without the influence of the illumination spectrum, nor the added weighting for the perceptual non-uniformity of Human vision (as is the case for the $\Delta E_{94}^{*}$ and $\Delta E_{00}^{*}$ , see (CIE, 2018)). Like ΔE , however, the *interpretation* of θ depends on the reference spectrum, that is the initial spectrum against which we compare the target spectrum. The greater the spectral angle, the further away from the reference spectrum the tested spectrum is. Conversely, if the angle is near zero then the spectra are similar. In the broader field of hyperspectral data analysis, such spectral features such can be used for classification of regions into different categories (e.g., (Vane and Goetz, 1988; Kruse et al., 2003)).

#### 2.2.1 Analysis of spectral angle distribution in an ROI

**FIGURE 2:**
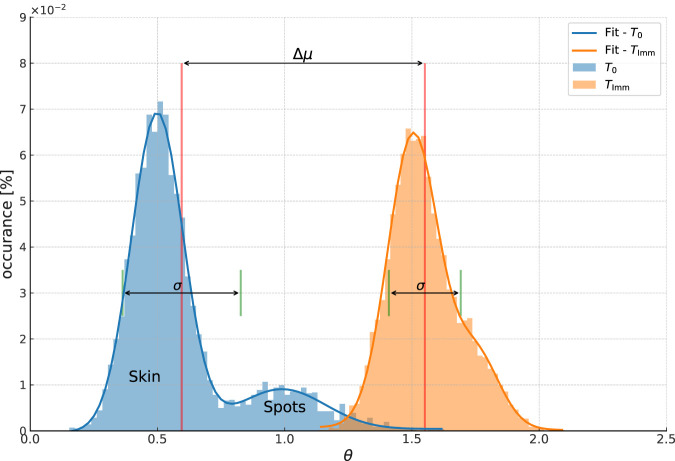
Conceptual example of the spectral angle distribution in a ROI. The blue peak is the distribution of spectral angles θ referenced to the average spectrum in the ROI at $T_{0}$ . Different features in the ROI give distinct values of θ . This results in multiple peaks in the distribution corresponding to each feature, the largest of which correspond to the base skin. Secondary peaks at higher spectral angles corresponds to features such as pigmented spots. The homogeneity of each feature determines the width of each peak, and the contrast between the features determines the distance between peaks. The orange peak shows the distribution of θ in the same ROI at $T_{\text{imm}}$ . The color shift of the product results in an increase in θ for the base skin. If the homogeneity of the bare skin increases, then the width of the main peak will decrease. At the same time, if the product brings the features closer in spectra to the base skin, then distinct peaks may begin to merge.

Aside from classification, another way in which to use the spectral angle is to analyze the appearance of spots and the homogeneity of the skin over a given area by looking at the distribution of spectral angles within the region. This is not dissimilar from the idea behind using the volume of the $L^{*}a^{*}b^{*}$ color distribution (Coxello index), but benefits from the increased sensitivity and specificity of the spectral angle as a parameter which measures the degree of difference between spectra. As a thought experiment, imagine that we take a bare skin hyperspectral image of a model, and that we select ROI on the model’s right and left cheeks. We then take the average spectrum from those regions as an estimation of the model’s average skin spectrum. If we take the spectral angle between each spectra in the ROI and this average spectrum and create a histogram of the number of pixels within a given range of spectral angles, we will get a distribution as shown in blue in Figure 2.

First, there will be a peak near zero containing all the pixels which have a spectrum near the average bare skin, labeled “Skin” in the figure. The peak will not be at zero, because the dot product between two spectra in Equation 1 must be positive valued, meaning that only pixels with a spectrum which is exactly the average will give a spectral angle of zero. The greater the typical difference between each spectrum and the average bare skin spectrum, the higher will be the mean value. At the same time, the wider the range of spectra in the ROI, the greater the width of the distribution. The peak position and the peak width therefore tell us about the homogeneity of the skin in the ROI.

At the same time, the areas which have distinctly different spectra from the average will give a larger spectral angle, creating one or more peaks to the right of the *skin* peak. We have labeled this peak as *spots* as it corresponds to the features such as pigmented spots, moles, etc. In principle, there will be one peak for each type of feature in the ROI, but in practice the ability to separate them will depend on their distance from one another and on the width of the feature spectral distribution, that is the variation of spectra within each feature. Importantly, the separation of the *skin* and *spots* peaks gives us information about the degree of contrast between the spots and the base skin.

Now let us consider what happens when we apply a makeup foundation. First, the foundation changes the apparent color of the bare skin. At the same time, the hope is that the spots become less visible, that is to say closer in spectrum (color) to the skin. If we again take the spectral angle between each spectra in the ROI and this average spectrum, we will get a distribution of angles like that shown by the orange-yellow curve in Figure 2. As the base skin color changed, this registers as an increase in θ relative to the bare skin spectrum, and so the change in the mean value of the spectral angle peak tells us about the magnitude of the spectral change caused by the makeup.

In addition, as the spots are now closer to the base skin color, the *spots* peak begins to merge with the skin peak. If we consider the limit of a perfect spot hiding product, there would be only one peak after product application, as the spots would be indistinguishable from the skin. The most powerful way to analyze this effect is to characterize the entire spectral angle distribution. Doing that, we can estimate parameters such as the area of spots before and after product application, the contrast between the spots and the base skin, the relative change in color of the spots and the skin, etc.

At the same time, this distribution is only for one, arbitrarily selected, region. Looking at a different region will give us a different distribution, say, for example, if the there are more spots in one area than in another. The degree of difference in the spectral angle distribution from one region to the next tells us about the evenness of the skin at the scale of the region size. So, in order to truly characterize the product effect we should in principle also study how the distribution changes over the face. We will come back to this topic later in our discussion.

For a first, simple, evaluation of makeup foundation coverage, on the other hand, we can attempt to use the width of the spectral angle histogram in one ROI. Functionally, we will focus on the number of different spectra over a large ROI and ignore the spatial evenness of the color. The standard deviation of the spectral angle histogram would be the natural choice to do this, but we would then not benefit from the additional knowledge of the shift in the mean of the distribution.

To make use of that information, we can use a different reference spectra to ask a slightly different question. We previously compared the spectra in the ROI at $T_{0}$ to the average bare skin spectra. The mean value of the distribution of this spectral angle relates to the number of colors in the ROI, i.e., the homogeneity of the bare skin and the distance between the skin spectral angle peak and the spots peak, that is the contrast between the spots and the skin. A larger value of $\mu_{\theta }$ indicates a wider distribution and/or greater contrast between the spot and the base skin. At $T_{\text{imm}}$ , we can repeat the same question and take the spectral angle with the average spectrum in the ROI at $T_{\text{imm}}$ . If the product improves the homogeneity of the skin color or decreases the spot contrast, then the mean spectral angle in the region will decrease relative to the value at $T_{0}$ .

We therefore have two measurements using two different reference spectra:

• Spectral homogeneity: by comparing to the average spectrum within the ROI at each time-point.

• Spectral change: by comparing to the average bare skin spectrum at $T_{0}$ .

In the next sections, we will apply this approach to the analysis of actual data from the HSI validation study. Doing so, we will see that the change in the spectral angle distribution caused by a makeup foundation looks like the theoretical example we considered so far and that we can indeed see a clear difference between the effect of different products.

#### 2.2.2 Spectral homogeneity

**FIGURE 3:**
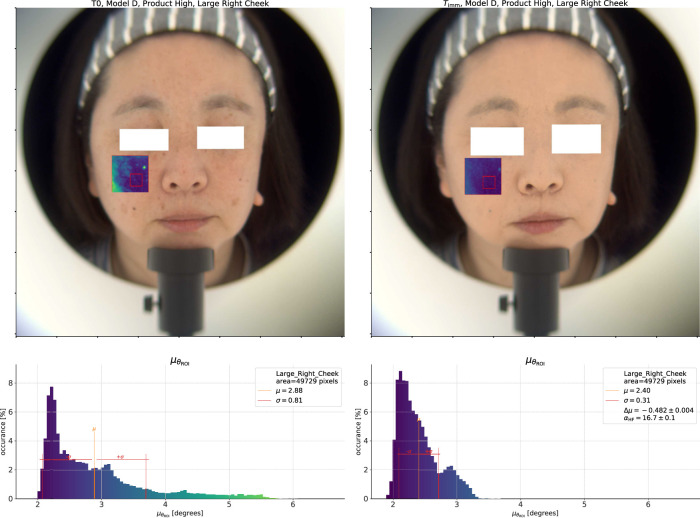
On the left, we show the $T_{0}$ measurement for the *High* coverage product test. The top pane shows the reconstructed color image with the calculated spectral angle overlaid on the “Large Right Cheek” ROI. The reference spectrum is the average within the smaller red rectangle. Below that, we show a histogram of θ within the ROI. We can see multiple peaks in the distribution corresponding to the base skin and different types of pigmented spots. On the right side is the same analysis after application of the *High* coverage product. The width of the spectral angle distribution has greatly decreased, and, where before there was a long tail, the overall distribution is now more compact.

So far we have discussed a theoretical model for coverage analysis based on the distribution of θ in a chosen ROI. We will now test this model on examples from the HSI validation study dataset. On the left side of Figure 3, we show an analysis of the spectral angle distribution on the right cheek of one model at $T_{0}$ (some example spectra from the same model are shown in Figure 1). Here we use the average spectrum from the 12 by 12 ${mm}^{2}$ ( 75×75 pixels^2^) “Right Cheek” ROI, outlined on the figure in red, as the reference spectrum to compute θ for each pixel in the 35 by 35 ${mm}^{2}$ ( 223×223 pixels^2^) “Large Right Cheek” ROI. We define each of these ROI by first locating 68 facial landmark points using the DLIB (King, 2009) Histogram-Orientation-Gradient (HOG) based face detector and then defining the ROI relative to the location of those landmarks.

We show these spectral angle results overlaid on a reconstructed color image (under CIE D65 illumination (ISO/CIE, 1999)) at the top of the figure. In the overlay, more blue values indicate lower spectral angles, while more yellow values indicate higher spectral angles, i.e., regions which are more different from the reference spectrum. At the bottom of the figure, we show a histogram of the spectral angles in the ROI with the same color map applied. Before application of any product, at $T_{0}$ , there is a large peak in the distribution at low values of the spectral angle corresponding to the base skin. Above that, there are smaller peaks corresponding to the different pigmented spots in the ROI. The average value of θ in the ROI, $\mu_{\theta }$ , is 2.88°, indicated on the plot by the solid black line, and the standard deviation, $\sigma_{\theta }$ , is 0.81°.

Now, we do the same analysis after application of the *High* coverage product using the average spectrum at $T_{\text{imm}}$ as the reference and show the result on the right side of Figure 3. First, we can note the clear made-up appearance of the model’s skin in the color image, and in the spectral angle overlay we can no longer see the large pigmented spot on the upper left (the model’s right side) which was visible before application. As for the effect on the θ histogram, whereas before application there were multiple peaks at θ>3.5 °, there are now almost no pixels which give a spectral angle in this range. The pixels with the most different spectra from the average, for example, the center of the small dark mole on the upper right, were at a spectral angle of between 5° and 5.5° before application $\left(\delta_{\text{skin}-\text{spot}}\approx 3^{{}^{\circ}}\right)$ , but these same pixels are now at a spectral angle of approximately 3° $\left(\delta_{\text{skin}-\text{spot}}=1^{{}^{\circ}}\right)$ . The difference in spot contrast is therefore about 66%. At the same time, the standard deviation of the total distribution has decreased to 0.31, and so too $\mu_{\theta }$ down to 2.40°. The difference of the mean values $\Delta \mu_{\theta }=\mu_{\theta }\left(T_{\text{imm}}\right)-\mu_{\theta }\left(T_{◦}\right)$ is −0.48°. As a negative value this indicates an *increase* in the homogeneity within this ROI after product application as the number of pixels with a spectrum which is far away from the average is lower after we apply the product.

**FIGURE 4:**
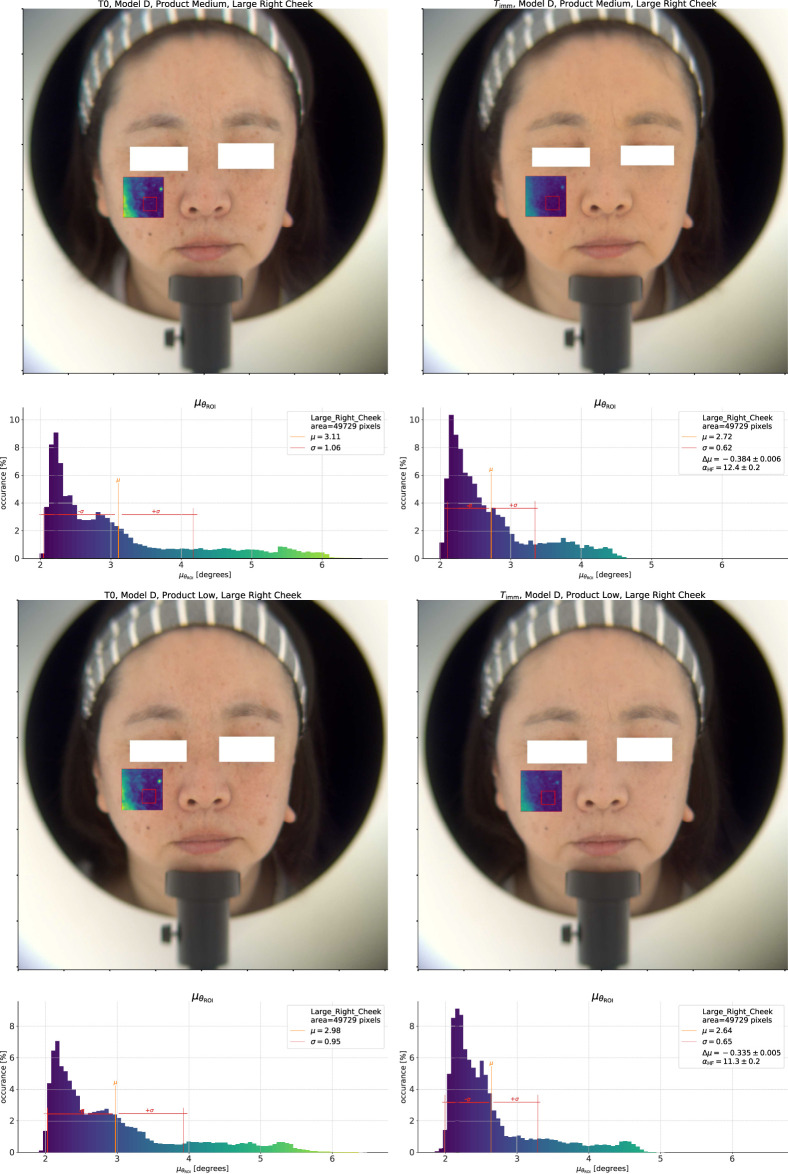
Top pane: Spectral homogeneity effect for the *Medium* coverage product. Bottom pane: *Low* coverage product.

The natural question which follows is whether we can differentiate the products using this approach, and to answer this question we show the same analysis for the *Low* and *Medium* coverage products in Figure 4. At first glance, the product effect on the θ distribution appears to be similar for the *Low* and *Medium* coverage products. The histogram for the *Medium* coverage product however shows a shorter tail and a smoother transition from 2° to 3° than the *Low* coverage product. In addition, the $T_{0}$ distribution of the Medium product is also different from the Low product $T_{0}$ measurement, which is due to the differences in bare skin condition of the model between tests. As a result, the difference $\Delta \mu_{\theta }$ is lower for the Medium product (−0.49°) than for the Low coverage product (−0.43°), indicating a greater improvement in homogeneity for the *Medium* coverage product.

#### 2.2.3 Spectral change–Color shift

**FIGURE 5:**
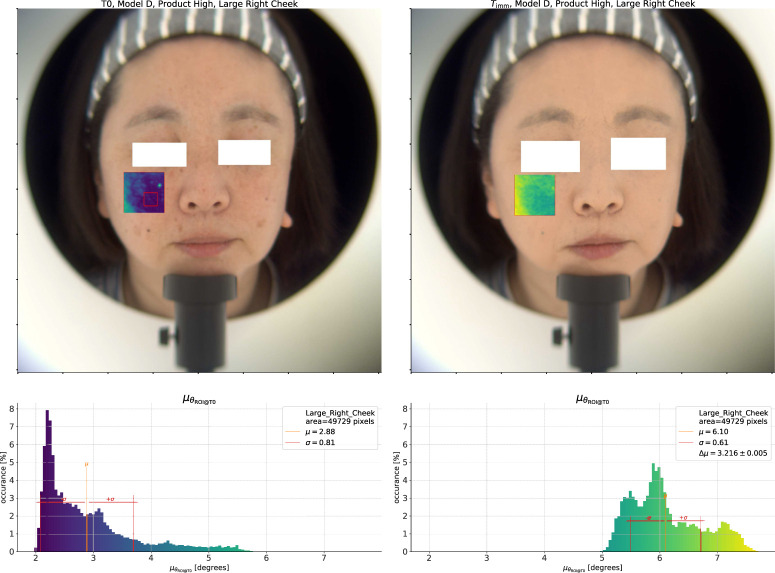
Analysis of the spectral change effect for the *High* coverage product. On the left, we show the $T_{0}$ measurement for the *High* coverage product test. The top pane shows the reconstructed color image with the calculated spectral angle overlaid on the “Large Right Cheek” ROI. Contrary to the previous analysis of the homogeneity in Figure 3, here we use the average within the smaller red rectangle at $T_{0}$ as the reference spectrum at all time points. Due to this, the histogram of θ within the ROI shows the change in spectral distribution over time (due primarily to the application of the product under test). As for the homogeneity analysis, we can also see the decrease in overall distribution width as all areas in the ROI move towards a new color.

Aside from the color homogeneity, we can also look at the color change. In Figure 5, we show a similar analysis of the spectral angle within a given ROI as in the last section. The key difference here is that instead of taking the average spectrum from the “Right Cheek” ROI at each time point as the reference spectrum, we instead use the average spectrum at $T_{0}$ as the reference at all time points. In the figure, we see that the distribution of the spectral angles at $T_{0}$ is the same as before, but the histogram of θ at $T_{\text{imm}}$ now differs. As the application of the product has modified all the spectra from what they were at $T_{0}$ , the entire distribution shifts towards higher values of spectral angle. The magnitude of this shift is similar to ΔE , and informs us about the total spectral change in the ROI. For the *High* coverage product shown here it is 3.22°.

At the same time, we can also see that the difference in spectral angle between the base skin and the spots has reduced, as evidenced by the decrease in $\sigma_{\theta }$ . In this case, the degree of change for the spots and the skin can give us insight into the product effect. For example, the lowest peak, corresponding to the skin, shifts from a spectral angle of ≈2.3 ° to around 5.5°, a difference of 3.2°. The features at the upper end of the $T_{0}$ distribution, however, shift from around 5.5°–7.3° at $T_{\text{imm}}$ , for a difference of 1.8°. We therefore see that the product covers the spots by shifting both them and the skin towards a new color, and the action on the skin is greater than on the spots.

**FIGURE 6:**
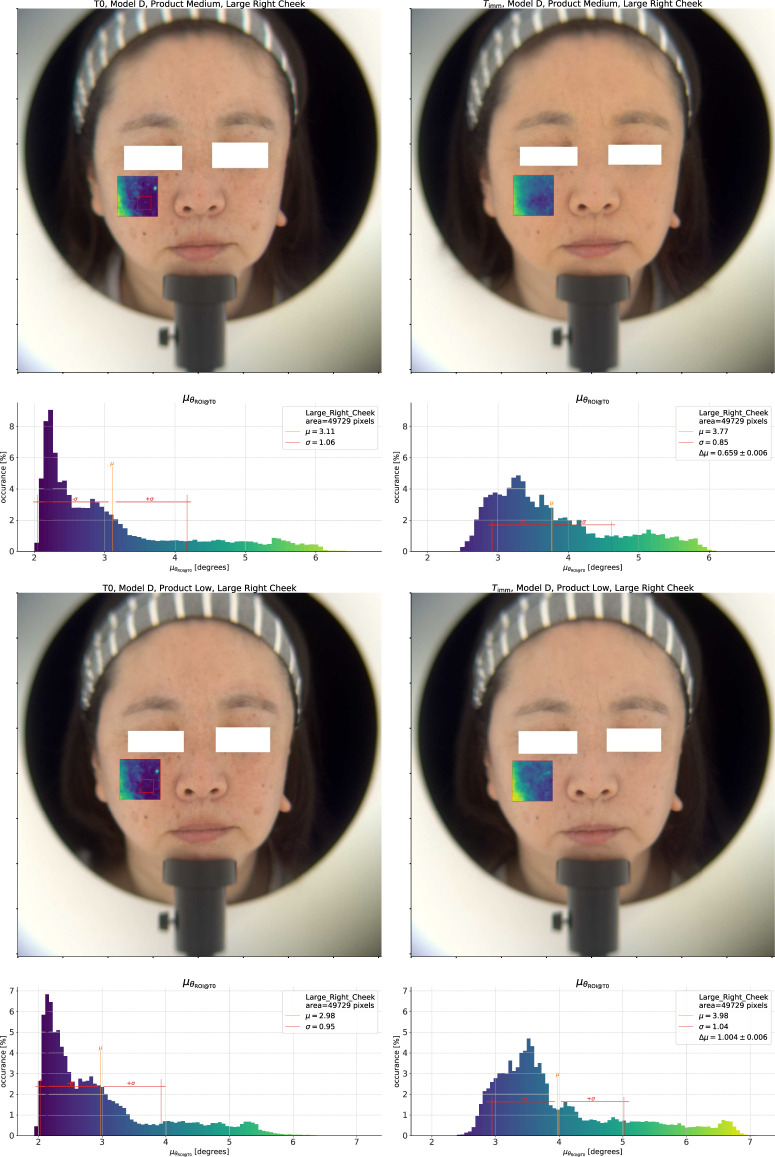
Top pane: Analysis of the spectral change effect for the *Medium* coverage product. Bottom pane: *Low* coverage product.

As before, it is important to compare the effect of different products to sharpen our understanding. We show the same color shift analysis for the *Medium* and *Low* coverage products in Figure 6. Focusing on the *Medium* result, we can immediately see that the total color change effect is lower than that of the *High* coverage product ( $\Delta \mu_{\theta }$ of 0.66° vs. 3.22° for the High coverage product). Again, we can look at the shift of the skin color vs. the spot color, and for the skin we see here a change from ≈2.3 ° to 3.5°, a change of 1.2°. At the same time, the spot in the upper right of the ROI went from around 6°–5.5°, giving a *decrease* of 0.5°. What we see in this case is that this product does modify the color of the base skin, but at the same time brings certain spots closer to the *original* skin color. This effect is in contrast to the action of the *High* coverage product, and we see that by analyzing the product effect on the spectral angle distribution we can gain insight into action of different products.

#### 2.2.4 Towards the evaluation of coverage

Our goal is to apply this methodology to a large dataset. Ideally, we would conduct a full analysis of the spectral angle distribution for each study sample, but doing so requires us to develop methods for automating the analysis (including the non-trivial handling of edge cases) and agglomerating results over samples. As a short term approach, we propose the creation of simple parameters which we extract from each spectral angle distribution in order to compare one product to another.

In this context, we propose a *Homogeneity Factor* defined using the spectral angle distributions at $T_{\text{imm}}$ and $T_{0}$ , referenced to the average skin spectrum at each time point. In this analysis, we define this as the percentage shift in the mean value of the spectral angle distribution:

$$\alpha_{HF}=100\left(1-\frac{\mu_{\theta_{\text{ROI}}}\left(T_{\text{imm}}\right)}{\mu_{\theta_{\text{ROI}}}\left(T_{0}\right)}\right)$$ (2)

where $\mu_{\theta }$ is the mean spectral angle at each measurement time. Defined in this way, $\alpha_{HF}$ will range from −∞ , indicating a drastic decrease in the homogeneity, to 100, indicating perfect homogeneity (all spectra in the ROI equal to the average).

We would like to note that, even if the improvement of the homogeneity of the skin over the face due to a product is the main driver of what is typically called coverage, it is certainly not the sole factor influencing this attribute. For one, we expect not only the homogeneity of the color, by which we mean the number of different colors present in the region, to play a leading role, but also the color evenness, or the change of color from area to area, to impact the perception of coverage. Since attributes like homogeneity and evenness are objective measurements, while coverage is a loaded term with significant differences in user preference and perception between groups, it is also therefore best to avoid giving the latter label to an instrumental measurement. We have therefore chosen to call $\alpha_{HF}$ the *homogeneity* factor as an indication of what it fundamentally measures. The degree to which $\alpha_{HF}$ correlates with the coverage is a question which we must address by comparing the instrumental results with sensory or consumer evaluation.

As a measure of the overall change in spectra within the region after product application, we will likewise define a *Spectral Shift Factor*, which for now we will simply define as the difference in mean spectral angle referenced to the average spectrum at $T_{0}$ :

$$\beta_{SF}=\mu_{\theta_{\text{ROI@T0}}}\left(T_{\text{imm}}\right)-\mu_{\theta_{\text{ROI@T0}}}\left(T_{0}\right)$$ (3)

Our expectation is that this parameter will follow closely the ΔE results, with the key difference that it is not influenced by the illumination and does not account for the transfer function of the human visual system. In the next section, we will apply these two new parameters to evaluate the products included in the HSI validation study.

## 3 Results

In the original validation study analysis, we ranked the products according to the values of ΔL , $\Delta a^{*}$ , $\Delta b^{*}$ , $\Delta E_{96}$ , and $\Delta I_{\text{Coxello}}$ (the change in $L^{*}a^{*}b^{*}$ color distribution width as a volume), between $T_{\text{imm}}$ and $T_{0}$ , averaged over models for each sub-test. We calculated $L^{*}a^{*}b^{*}$ coordinates from the measured spectra for the CIE 1964 $10^{{}^{\circ}}$ observer (Stiles and Burch, 1959; Speranskaya, 1959; ISO/CIE, 2019) under D65 illumination (ISO/CIE, 1999). In order to test the new spectral angle based parameters for coverage analysis, we will calculate $\alpha_{HF}$ and $\beta_{SF}$ for each product and model from one validation sub-test (one repetition, for one operator). We will also report $\Delta E_{96}$ and $\Delta I_{\text{Coxello}}$ , to compare our new parameters for coverage evaluation with past methods.

For each model, we define a “Large Right Cheek” ROI of 35 by 35 mm as our working ROI, and a 12 by 12 mm “Right Cheek” ROI as the source of the reference spectrum to compute θ for each pixel in the working ROI. We define both of these ROI relative to the location of each model’s facial features using automatic landmark recognition, as we summarized previously. As we extract the ROI automatically, we of course have both the right and left cheek regions available. The results from the two sides are comparable, but individual models do show differences between sides depending on their individual arrangement of spots and other skin imperfections. This is because both the initial skin homogeneity and the ROI selected affect the results. We will discuss these points later and at the moment will focus on only the right side data for brevity.

After defining the ROI, we calculate the spectral angle for each pixel in the working ROI for each time point. The spectral angle with reference to the average spectrum in the “Right Cheek” at that time point gives us $\theta_{\text{ROI}}\left(t\right)$ , and the spectral angle referenced to the average spectrum in the “Right Cheek” at $T_{0}$ gives us $\theta_{\text{ROI@T0}}\left(t\right)$ . We then calculate the mean value of the spectral angle distributions in the working ROI, as in Section 2.2.2, to obtain $\mu_{\theta_{\text{ROI}}}\left(t\right)$ and $\mu_{\theta_{\text{ROI@T0}}}\left(t\right)$ , from which we calculate $\alpha_{HF}$ and $\beta_{SF}$ according to (Equations 2, 3).

**FIGURE 7:**
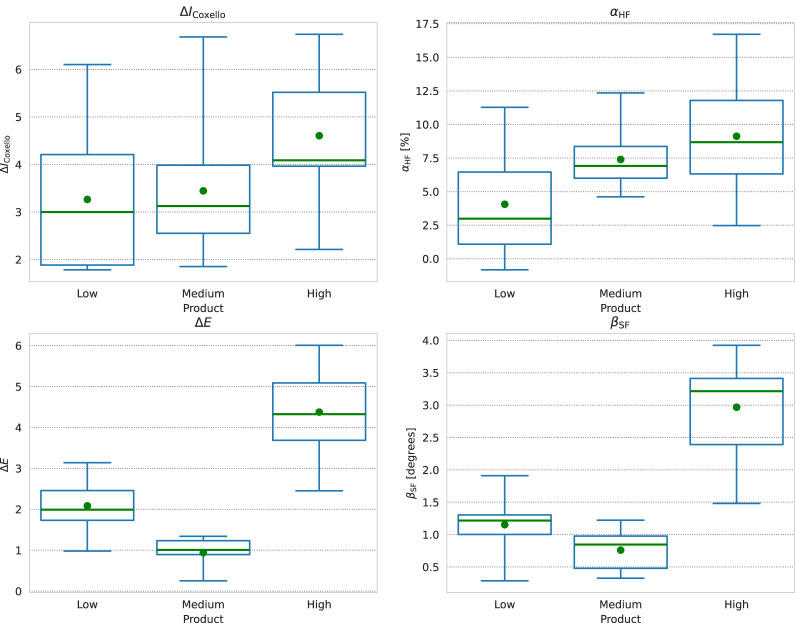
Box plots of (from top-left, clockwise) $\Delta I_{\text{Coxello}}$ , $\alpha_{HF}$ , $\beta_{SF}$ , and $\Delta E_{96}$ . We plot the values from the “Large-Right-Cheek” ROI by product in abscissa. The box extends from the first quartile $\left(Q_{1}\right)$ to the third quartile $\left(Q_{3}\right)$ . The solid colored line in the box denotes the median value $\left(Q_{2}\right)$ of the set, while the circle marks the mean. The whiskers denote the full range of the data. In this plot, we inverted the $\Delta I_{\text{Coxello}}$ results (positive value indicates an increase in homogeneity) to make it easier to directly compare with the $\alpha_{HF}$ results.

We show box plots of the $\Delta I_{\text{Coxello}}$ , $\alpha_{HF}$ , $\beta_{SF}$ and $\Delta E_{96}$ results over the nine models of one validation sub-test in Figure 7. In these plots, the box extends from the first quartile $\left(Q_{1}\right)$ to the third quartile $\left(Q_{3}\right)$ of the distribution, and the solid colored line in the box denotes the median value $\left(Q_{2}\right)$ of the set. The dashed line, on the other hand, shows the mean value, which is more strongly influenced by extreme values in the set, and the whiskers denote the full range of the data. To make it easier to directly compare it to the $\alpha_{HF}$ results, we have reported a decrease in $\Delta I_{\text{Coxello}}$ , indicating an increase in the color homogeneity in the ROI as a positive value.

As we expect, the overall trend in the two parameters is the same. The width of the distribution of $\Delta I_{\text{Coxello}}$ results are higher than those of the $\alpha_{HF}$ results for all three products, and the interquartile ranges $\left(Q_{1}-Q_{3}\right)$ overlap for the *Low* and *Medium* coverage products. In particular, these two products give almost the same median value of $\Delta I_{\text{Coxello}}$ , and only a slightly different mean.

The $\alpha_{HF}$ results, in contrast, show a reduced distribution width, and the first to third quartile $\left(Q_{1}-Q_{3}\right)$ ranges of the *Low* and *Medium* product results are smaller than for $\Delta I_{\text{Coxello}}$ . We also see that the median and mean values of these two products no longer overlap, indicating that we can differentiate these two products using the homogeneity factor. We give a summary of the mean results by parameter in Table 2.

**TABLE 2:** Summary of results for the *Large-Right-Cheek* ROI after product application. We report each value as the mean ± the standard deviation of the result over samples (models).

Product	Time	ΔE	$\Delta I_{\text{Coxello}}$	$\alpha_{\text{HF}}$	$\beta_{\text{SF}}$
Low	$T_{\text{imm}}$	2.08 ± 0.64	3.26 ± 1.68	4.06 ± 3.98	1.15 ± 0.49
Medium	$T_{\text{imm}}$	0.94 ± 0.38	3.44 ± 1.40	7.39 ± 2.26	0.76 ± 0.31
High	$T_{\text{imm}}$	4.38 ± 1.10	4.61 ± 1.40	9.13 ± 4.27	2.97 ± 0.83

The $\beta_{SF}$ and $\Delta E_{96}$ parameters follow one another, as expected, and we can distinguish the three products in terms of their spectral (and color) change effect. The *High* coverage product gives the greatest spectral change and the *Medium* coverage product gives the least spectral change. This is interesting as it shows that the perceived coverage is distinct from the magnitude of the color change caused by the product. There are some differences in the distribution widths between $\Delta E_{96}$ and $\beta_{SF}$ which likely relate to the relationship of a given spectral difference to the apparent color shift under a D65 illumination.

Next, we evaluated the statistical significance of the difference in measured values between products using the Python Statsmodel (Seabold and Perktold, 2010) library implementation of the Analysis of Variance (ANOVA) algorithm (Fisher, 1921) to determine if we can reject the null hypothesis (that no difference exists), followed by pairwise Multiple Comparison of Means (Tukey HSD) (Tukey, 1949) to group the products into statistically distinct subsets (if warranted). Here we used a test significance of 0.05 for both the ANOVA and the Tukey HSD tests. We give the result of this statistical analysis in Table 3. We find three distinct and consistent statistical groups for $\Delta E_{96}$ and $\beta_{SF}$ (*Medium*, *Low*, *High*), while we find no significant difference between the $\Delta I_{\text{Coxello}}$ results for the three products.

**TABLE 3:** Statistical analysis of products grouped by $\Delta E_{96}$ , $\beta_{SF}$ , $\Delta I_{\text{Coxello}}$ , and $\alpha_{HF}$ .

Parameter	$N_{\text{Groups}}$	Ranking	Results	Significant	*p*-value
$\Delta E_{96}$	3	M,L,H	0.94, 2.08, 4.38	Y	$5.4\cdot 10^{-9}$
$\beta_{SF}$	3	M,L,H	0.76,1.15,2.97	Y	$4.8\cdot 10^{-8}$
$\Delta I_{\text{Coxello}}$	1	(L,M,H)	(3.26,3.44,4.61)	N	$1.4\cdot 10^{-1}$
$\alpha_{HF}$	2	L, (M,H)	4.06, (7.39,9.13)	Y	$2.1\cdot 10^{-2}$

For each parameter, we show the ANOVA *p*-value results, as well as the Multiple Comparison of Means grouping analysis. We list the product with the smallest value first, with parentheses to indicate groups, and give the means for each product in the “Results” column in order of the grouping. The “Significance” column indicates if we can dismiss the null hypothesis (no difference in the products) at the test-threshold of 5%.

For $\alpha_{HF}$ , on the other hand, we find that the difference between the products is statistically significant, but the multiple comparison of means analysis classifies the *High* and *Medium* coverage products in the same group, due to the overlap of the $\left(Q_{1}-Q_{3}\right)$ ranges of each combined with the sample size. The *p*-value for the $\alpha_{HF}$ results is $2.1\cdot 10^{-2}$ vs. $1.4\cdot 10^{-1}$ for $\Delta I_{\text{Coxello}}$ , indicating that $\alpha_{HF}$ offers greater statistical power in this study.

The grouping results depend on the order in which we construct the samples, however, indicating that the model-to-model variability in the homogeneity effect is important. This raises the question of whether the variability between models is a inherent feature of attempting to correlate coverage with spectral homogeneity, or if there are some confounding effects for which we are not accounting. We will discuss this point further in the next section.

## 4 Discussion

**FIGURE 8:**
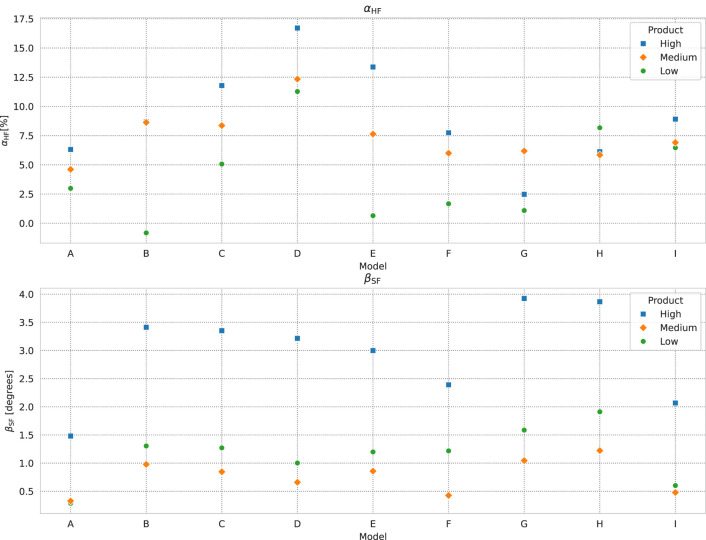
Plot $\beta_{SF}$ , and $\alpha_{HF}$ results by model for each product. The ranking between the products by homogeneity increase is consistent with the coverage for two thirds of the samples. The ranking by spectral change is the same for all models but one. See text for further discussion.

We find that, when averaged over all models, the sensory coverage evaluation correlates well with the change in spectral homogeneity after application, parameterized by $\alpha_{HF}$ . On a model-to-model basis the effect is more varied, however, and it is therefore worth looking at the results for each model. We show a complete analysis of the spectral homogeneity for the models who gave the maximum, minimum and (closest to the) mean effect for each product in the Supplementary Material, and we plot the $\alpha_{HF}$ and $\beta_{SF}$ results for each product by model in Figure 8. We see from these plots that, while the magnitude of $\alpha_{HF}$ varies from model to model, the relative ranking of the *High*, *Medium*, and *Low* coverage products is consistently in that order for two thirds of the sample set (6 of 9). For model “G” we see the greatest effect for the *Medium* product, while for model “H” the *Low* coverage product gives the best effect. For model “B”, on the other hand, the *Medium* and *High* coverage products give almost the same effect, and the *Low* coverage product decreases the spectral homogeneity.

If it seems difficult to envision that the addition of a color-correcting film on top of the skin can *decrease* the homogeneity, it is important to keep in mind that the homogeneity we are talking about is the number of *spectra* in the region and their degree of difference. This determines, but is distinct from, the number of colors in the area and the ΔE between them, both of which depend on the illumination spectrum.

**FIGURE 9:**
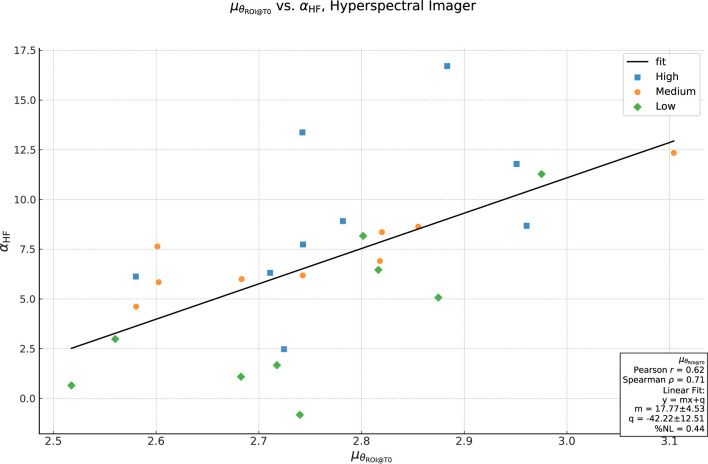
Plot of correlation between $\mu_{\theta }\left(T_{◦}\right)$ in abscissa vs. $\alpha_{HF}$ in ordinate. The black line shows the result of a least-squares linear fit to the full dataset.

To further understand the $\alpha_{HF}$ results across models, we note that the magnitude of $\alpha_{HF}$ varying from model-to-model is in line with the fact that $\alpha_{HF}$ measures the *change* in spectral homogeneity in a region. Due to this, the magnitude of the change will depend on the level of inhomogeneity in the area at $T_{0}$ , e.g., the number and visibility of spots on the model’s skin, *etc.* To check this relationship, we plot $\alpha_{HF}$ in ordinate vs. $\mu_{\theta }\left(T_{0}\right)$ in abscissa in Figure 9. Although the dependence is not linear, the correlation coefficient result r=0.6 indicates a relation between the degree of homogeneity change and the initial homogeneity. Along these lines, $\alpha_{HF}$ also implicitly depends on what region we chose, an important point which we will return to later.

**FIGURE 10:**
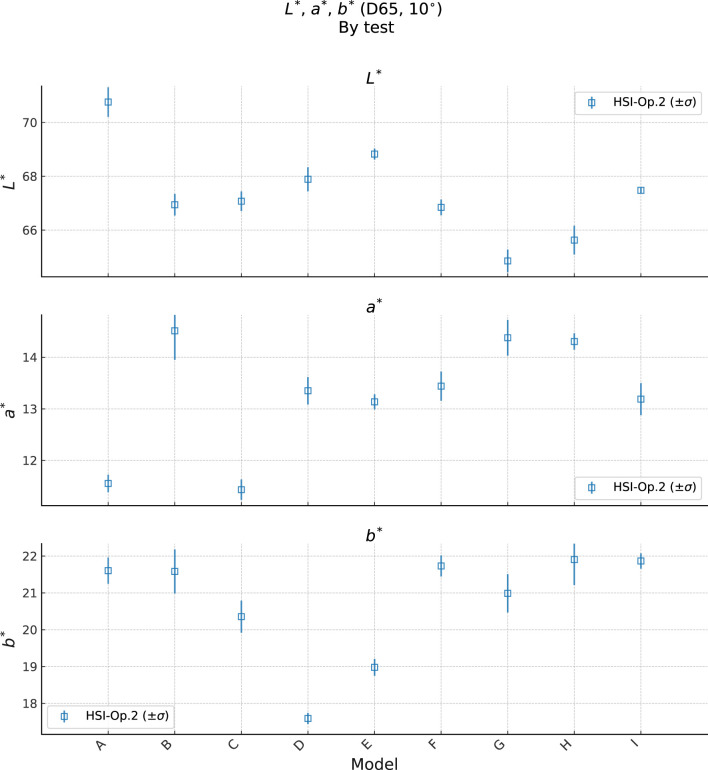
Plot of bare skin $L^{*}a^{*}b^{*}$ values for each model. We have take the average color over the working ROI, averaged over the independent $T_{0}$ measurements from each product. The error bars indicate the standard deviation over the three $T_{0}$ measurements taken at different times.

The fact that the ordering (ranking) of the products by $\alpha_{HF}$ changes for some models may indicate a dependence on the model’s skin tone relative to the product shade. First, we note that the three models where the $\alpha_{HF}$ ranking changed also showed the greatest values of $\beta_{SF}$ for the *High* coverage product. This may indicate a poor match between these model’s skin tone and that product. To further investigate this, we show a plot of the average bare skin color of each model in Figure 10. Here we see that the model “G” had the lowest $L^{*}$ value, and this may account for the poor effect of the *High* coverage product, which is in a light shade (see the Supplementary Material for a full analysis of this model’s result). Likewise, model “H” had the second lowest $L^{*}$ value, with a correspondingly poor result for the *High* coverage product, but this model also had among the highest $b^{*}$ value and $a^{*}$ values in the set, which may be responsible for the good effect from the *Low* coverage product. Finally, model “B” had the highest $a^{*}$ value, and showed a better than typical result for the *Medium* coverage product (in comparison to *High*).

It is important to keep in mind here that the $L^{*}a^{*}b^{*}$ values occupy a three dimensional color space and so the correlation between $\alpha_{HF}$ and any single coordinate does not fully characterize their relationship. Fully understanding the product effect as a function of the bare skin color and the product shade requires us to consider their relative location in the $L^{*}a^{*}b^{*}$ color space and the distance (ΔE) between them. We intend to further explore the correlation between these two parameters and the homogeneity result in the future.

Looking at the spectral shift, $\beta_{SF}$ , we see that the ranking is the same for all models except model “H”, for whom the *Medium* coverage product gave a slightly higher spectral change than the *Low* coverage product. This model had the lowest $a^{*}$ value of the set, and we note that each of the $\beta_{SF}$ results for this model are the lowest for their respective products. Along these lines, a key advantage of $\beta_{SF}$ in contrast to ΔE is that a given ΔE is true under one illumination, whereas the spectral shift $\beta_{SF}$ is true under all possible illuminations. If we accept that the product coverage effect depends on the matching of the product shade with the model skin tone, then working with the spectral matching rather than illumination-dependent $L^{*}a^{*}b^{*}$ color matching can be an important consideration.

**FIGURE 11:**
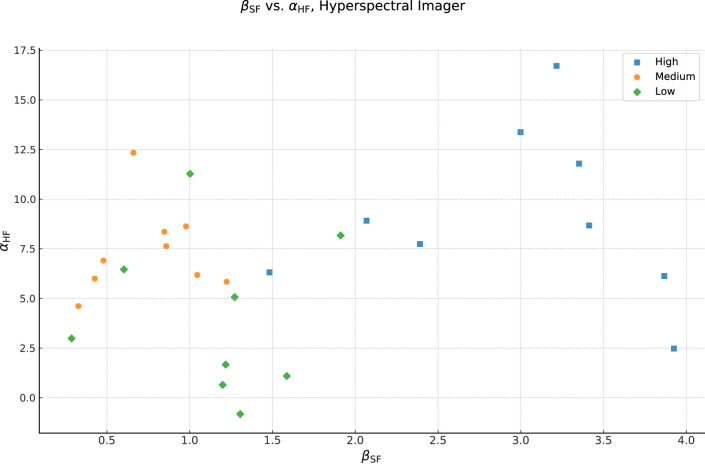
Correlation of the $\alpha_{HF}$ results in ordinate vs. the $\beta_{SF}$ results in abscissa, labeled by product. Functionally, this plot is a map of foundation homogeneity vs. color change effect. Square markers indicate the *High*, circle markers the *Medium*, and diamond markers the *Low* coverage product. The *High* coverage product gives a significant homogeneity improvement, but also a strong spectral (color) change. The *Medium* coverage product gives a good improvement in homogeneity with the least spectral shift, this makes it clear that the coverage and the color change are distinct effects. The *Low* coverage product gives the least homogeneity increase while also changing the spectra more than the *Medium* coverage product on average. However, we can also see that there are cases where $\beta_{SF}$ or $\alpha_{HF}$ are higher or lower than the typical result for that product, perhaps indicating a particularly good or poor match between the foundation shade and model’s skin tone.

Considering that, it is interesting to look at the relationship between $\alpha_{HF}$ and $\beta_{SF}$ , which we show in Figure 11, in order to better characterize these three products. Although there is significant overlap between the groups due to the model-to-model variability, we can see the emergence of three distinct product categories. The *High* coverage product is concentrated in the upper right side of the plot, indicating that this product gives both a strong spectral change (depending on the direction of the effect and the user preference we can characterize this as a color correction) and increase in homogeneity. The *Medium* coverage product, on the other hand, is at the upper left of the plot, indicating a weaker spectral change effect, but with good homogeneity increase. Finally, the *Low* coverage product is at the bottom left of plot, indicating a middling spectral effect (stronger than the *Medium* product) with a smaller increase in homogeneity.

As we discussed so far, these results are when applied to the average bare-skin $L^{*}a^{*}b^{*}$ values (corresponding to one spectrum under fixed D65 illumination) of the models in this dataset and for the product shades used in this test. The evidence of non-negligible dependence of the product coverage result on the relationship between the model skin tone and the product shade suggests that we consider a change in test design for makeup foundation screening, as, ideally, we should separate the product and shade variables.

As a concrete example, the current study design applied 3 products to 9 models of various skin tones, giving 9 samples per product with minimal control over shade matching effect. A possible future test design could instead apply 3 products to 3 models of the same skin tone over 3 shade groups to give 9 samples per product, grouped by shade. We could then construct a relative ranking between products and map them for a constant panel of models. As the magnitude of the homogeneity change varies from model to model, however, it is difficult to conclude an absolute coverage value for a product independent of its application to at a specific model (just as an object *under a specific light* determines color, a product *on a given skin* determines absolute coverage).

This leads us back to a previous point, that the magnitude of the homogeneity effect depends on where we look, that is the ROI which we use for the measurement. Consider simply that the result from an ROI which includes a strongly pigmented spot and another nearby ROI which does not include the spot will be different. Concretely, if we re-do our analysis over the validation dataset using the smaller “Right Cheek” region as our working ROI, we find that the magnitudes of the $\alpha_{HF}$ effect are lower on average (roughly a factor of 2) and that there is less difference between the mean effect of the *Medium* and *Low* coverage products. This raises the natural question of which ROI is the most representative of the “true” coverage effect. At the same time, we know that the perception of coverage is likely related not only to the improvement in homogeneity, but also to the apparent texture or evenness of the skin. The $\alpha_{HF}$ parameter does not measure this aspect, and although it may not be obvious at first, these two issues of texture and measurement area are interrelated.

In order to answer the question of which ROI is the correct one in which to evaluate the coverage, we should in principle repeat our analysis for every possible ROI and compare the result from each, that is to say that we should effectively look everywhere on the face and at all scales. Practically, we can do this by drawing a large enough number of ROI of random size at random positions throughout the working region (such as the full cheek). The different sizes of these ROI allows us to check the dependence of the homogeneity change on the size scale, while the randomized location accounts for the variability of features, such as spots, within the face. The variance of results (whether homogeneity, color, etc.) across ROI at a selected size scale tells us about the texture or evenness of the skin at that scale. For example, if we have a model with dense freckles, the variance of color for ROI of the same size as the freckles will be much higher than at a larger size ROI where we would be taking the average over a number of freckles and areas of skin.

As a final note, in this study, we explored only a univariate analysis approach. Given the relationship between the initial skin color and homogeneity and the homogeneity and color effect of the products, this data will benefit from an approach using multivariate analysis. In addition, while for this first attempt we used the mean value of the spectral angle to construct a simple homogeneity parameter, we expect analysis of the complete distribution of spectral angles within each ROI to provide greater discrimination and explanatory power. For this reason, we are currently working on combining analysis of the spectral angle histograms and additional statistics calculated from them with a multi-ROI analysis of the type discussed above.

## 5 Conclusion

In this study we have started unravelling the complex topic of makeup coverage. In the fullest sense, coverage is a perceived attribute, but from a purely optical perspective, we expect that the perception of coverage for makeup products comes from the color change caused by the product, the change in color homogeneity and evenness over the face after application, and the ability of the product to hide spots and other blemishes. As the previous instrumental measurements do not consistently correlate with coverage in a way which allows us to compare one product to another, we have begun exploring the new parameters and analysis methods made available by hyperspectral imaging.

As a starting point, we defined a homogeneity factor $\alpha_{HF}$ as a percentage change in mean spectral angle over an area after application of the product $\left(T_{\text{imm}}\right)$ , referenced to the average spectrum in the area. This parameter serves as a measurement of the change in the homogeneity of the spectra, which we expect to be one component of coverage. We likewise defined a spectral shift factor $\beta_{SF}$ , taking the absolute change in mean spectral angle over an area, referenced to the average spectrum in the area before product application $\left(T_{0}\right)$ . This parameter indicates the degree of spectral change after product application, which characterizes the color change effect of the product.

To test these new parameters and the overall analysis method, we applied them to an existing dataset, containing data for three makeup foundation products of different coverage levels (based on sensory evaluation) applied to nine models. We found that $\alpha_{HF}$ correlates with the sensory ranking of coverage when averaged over the dataset, and the distinct effect of the three products is clear on a model-by-model basis. Similarly, the parameter $\beta_{SF}$ correlates well with the visible color change induced by the product, but, unlike ΔE , is constant under all illuminations. By comparison of the homogeneity change $\left(\alpha_{HF}\right)$ and spectral change $\left(\beta_{SF}\right)$ for each product applied to each model, we find that we can map the three products into distinct groups by effect. Nevertheless the homogeneity factor $\alpha_{HF}$ does not fully describe coverage, and we find significant variability in the effect of each product from model to model.

This variability in product effect manifests as both a change in the magnitude of effect from model to model with the relative ranking between the products preserved, and as a change in the ranking between products for some models. In the latter, we see hints that this is the influence of the relative color different between the model’s skin tone and the product shade. As a next step in understanding this, we propose to assign each model a skin tone classification according to their average skin color, and look at the homogeneity effect vs. skin tone vs. product shade. If we indeed see a relationship between the model’s skin-tone and the homogeneity effect then this has clear implications for the design of future tests.

Likewise, the change in effect magnitude between models relates to the simple fact that the degree of homogeneity change which can occur depends on the starting inhomogeneity, which also implies that the measured effect depends on the selected ROI. This goes back to the general issue we face of the systematic uncertainty in evaluation results due to ROI selection. In addition, our homogeneity parameter is a function of the number of different spectra (colors) in the region and does not take into account the spatial distribution of the color. Intuitively, the evenness or texture is likely an important part of the perceived coverage effect, and we should therefore include it in our evaluation of makeup coverage. We can tackle both of these points by employing an analysis over the space of all possible ROI at varying size scales, combined with analysis of the full distribution of spectral angles within each ROI.

We also foresee the extension of this coverage analysis to include the lasting of the coverage over time. From preliminary data over multiple time points, we know that we can see the change of the homogeneity and color shift effects over time. There are some issues regarding the design of a such a study which we must address, such as how to accelerate the makeup wear in a way which does not bias the results, but from an analysis perspective, the main work which remains it is to improve our basic analysis of coverage in the directions which we have outlined here.

## Data Availability

The raw data supporting the conclusions of this article will be made available by the authors, without undue reservation.
