# Supplementary material for: A new method for the evaluation of makeup coverage using hyperspectral imaging
Source: Front Chem. 2024 Sep 10;12:1400796. doi: 10.3389/fchem.2024.1400796 (PMC11422780; doi:10.3389/fchem.2024.1400796)
Supplement: Supplementary file 1 [file DataSheet1.PDF]

Supplementary Material

1 SUPPLEMENTARY TABLES AND FIGURES

In this appendix we present selected examples from the dataset. For each product we show an analysis of the T_0 and T_{imm} images for the model closest to the *mean* change in α_{HF}. We calculate the color images from the HSI spectra in each pixel under D65 illumination. The working ROI is outlined in red, and the smaller ROI used as the source of the reference spectrum is likewise outlined on each image. Below each image we present a histogram of α_{HF} in the ROI, and we show the two histograms on the same scale to facilitate the comparison between before and after product application. We also indicate the mean value of $\mu_{\theta_{\mathrm{ROI}}}$ within the ROI and the change between the time points in the plot legend.

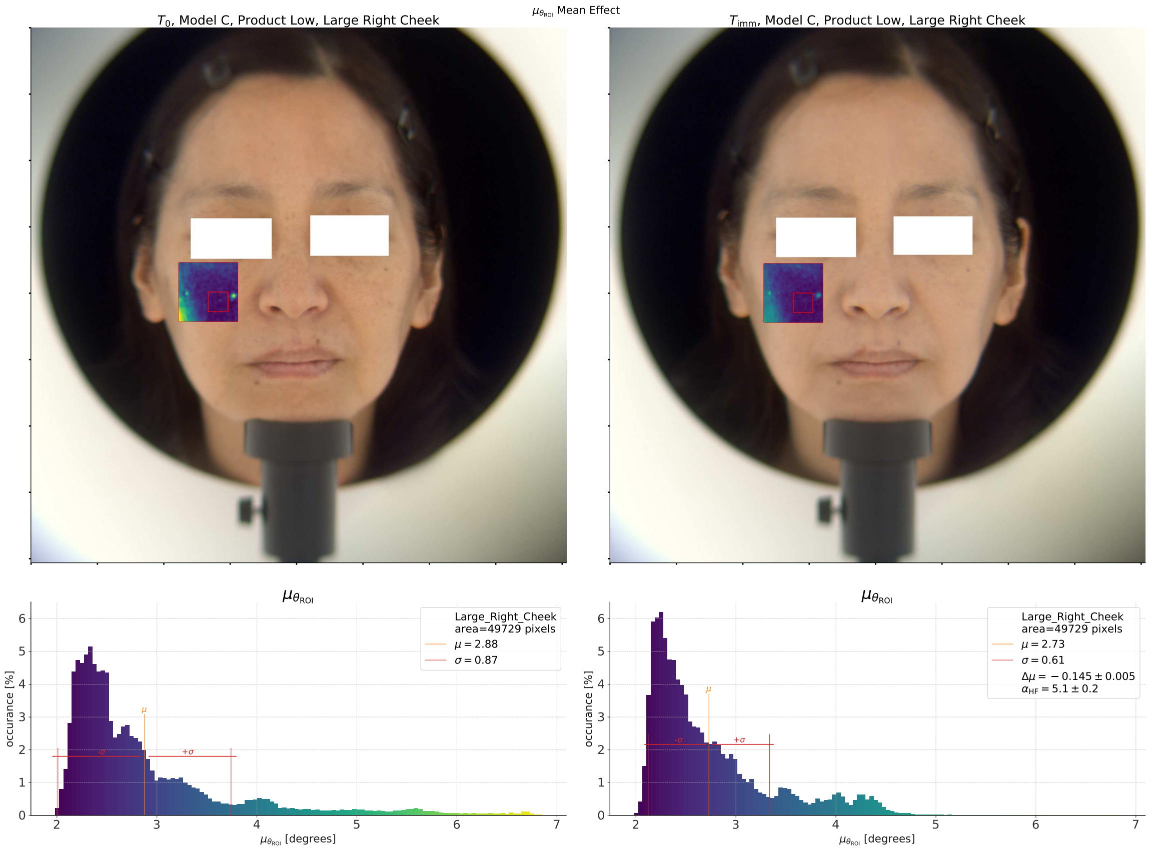

Figure S1. Data Example: Mean $\mu_{\theta_{\mathrm{ROI}}}$ result for product *Low*.

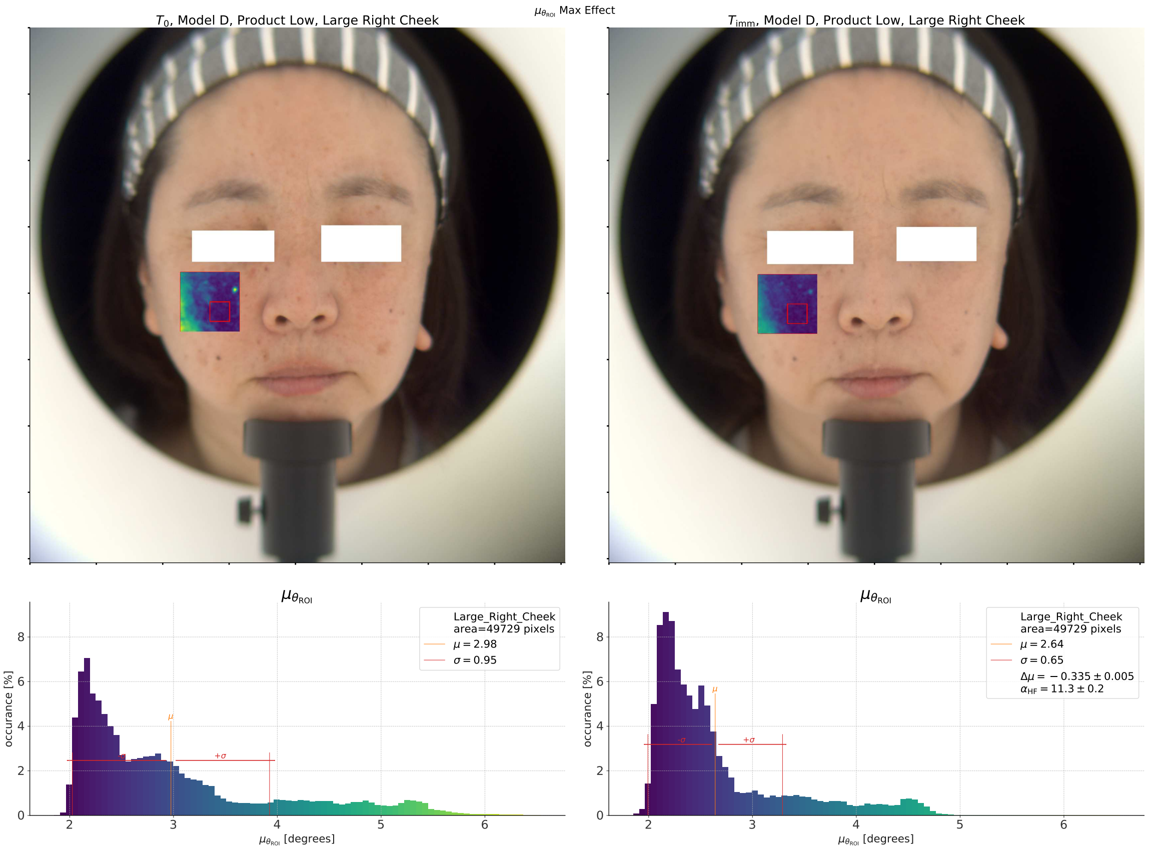

Figure S2. Data Example: Max $\mu_{\theta_{\mathrm{ROI}}}$ result for product *Low*.

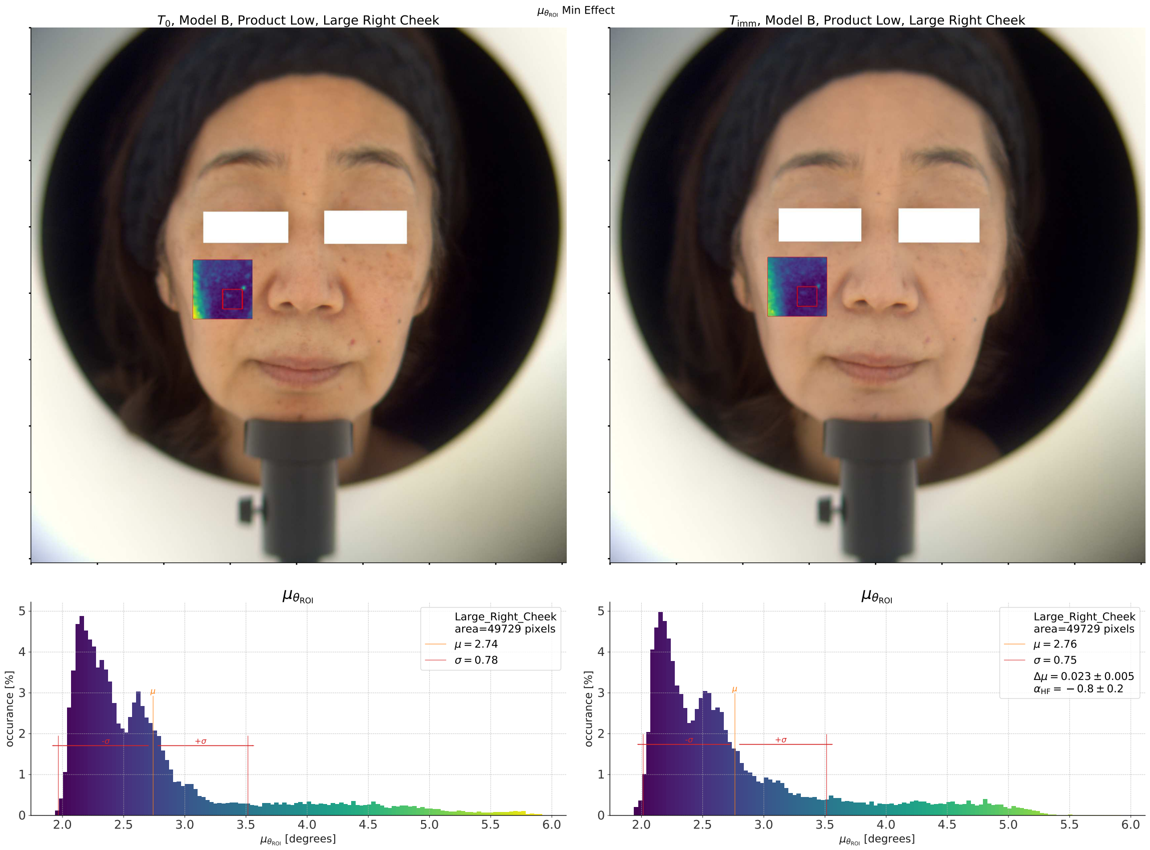

Figure S3. Data Example: Min $\mu_{\theta_{ROI}}$ result for product *Low*.

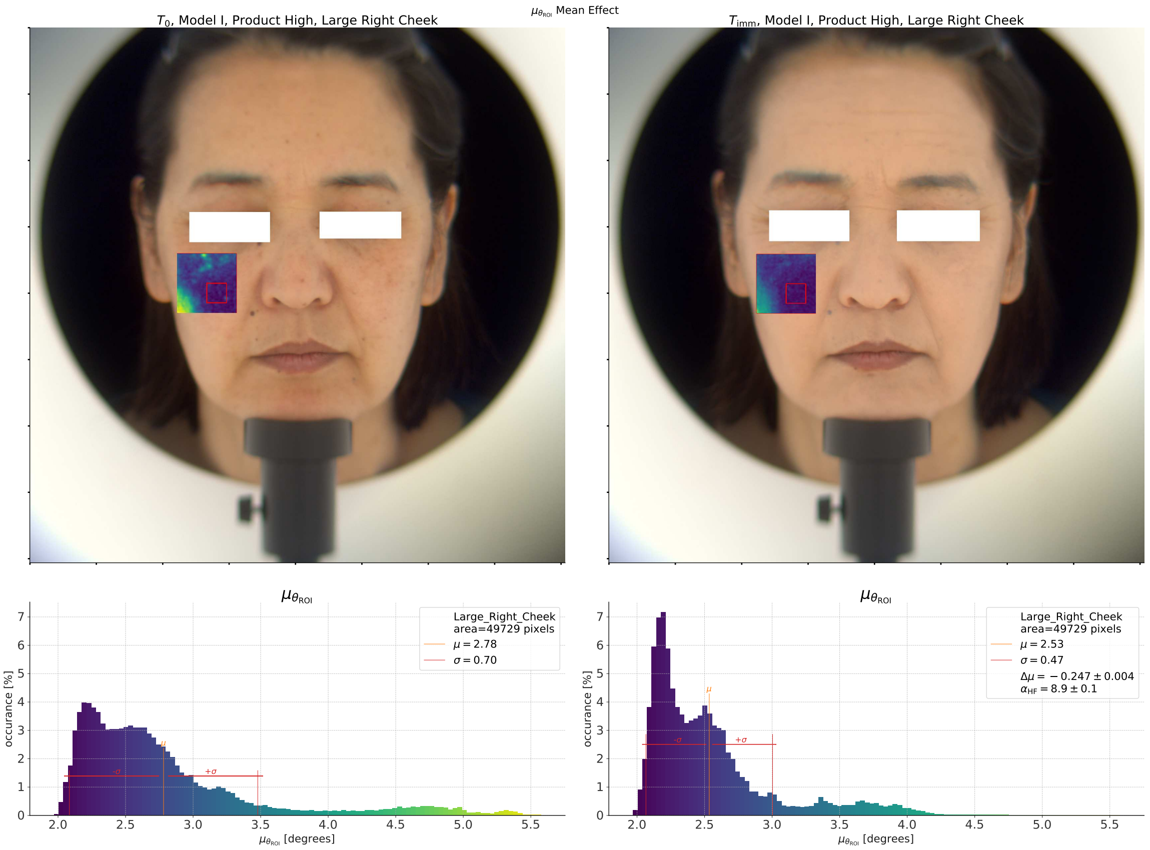

Figure S4. Data Example: Mean $\mu_{\theta_{\rm ROI}}$ result for product *High*.

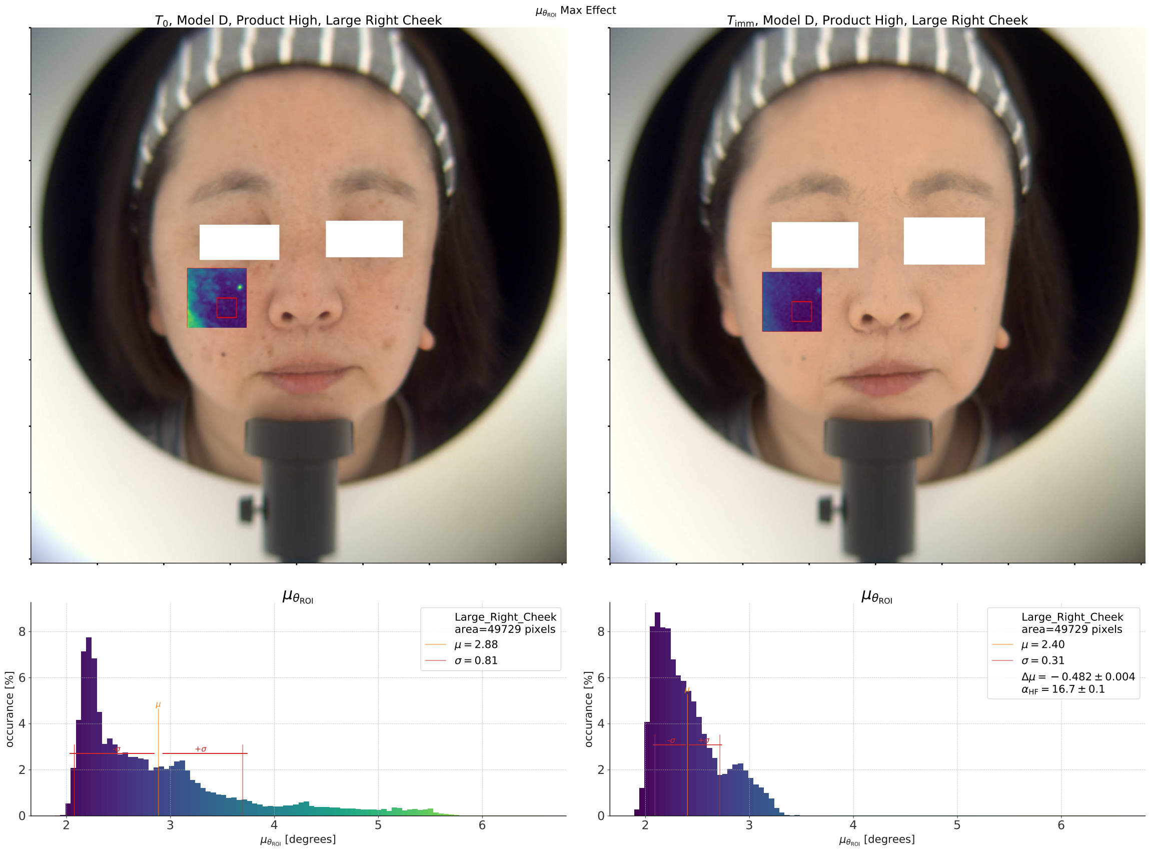

Figure S5. Data Example: Max $\mu_{\theta_{\mathrm{ROI}}}$ result for product *High*.

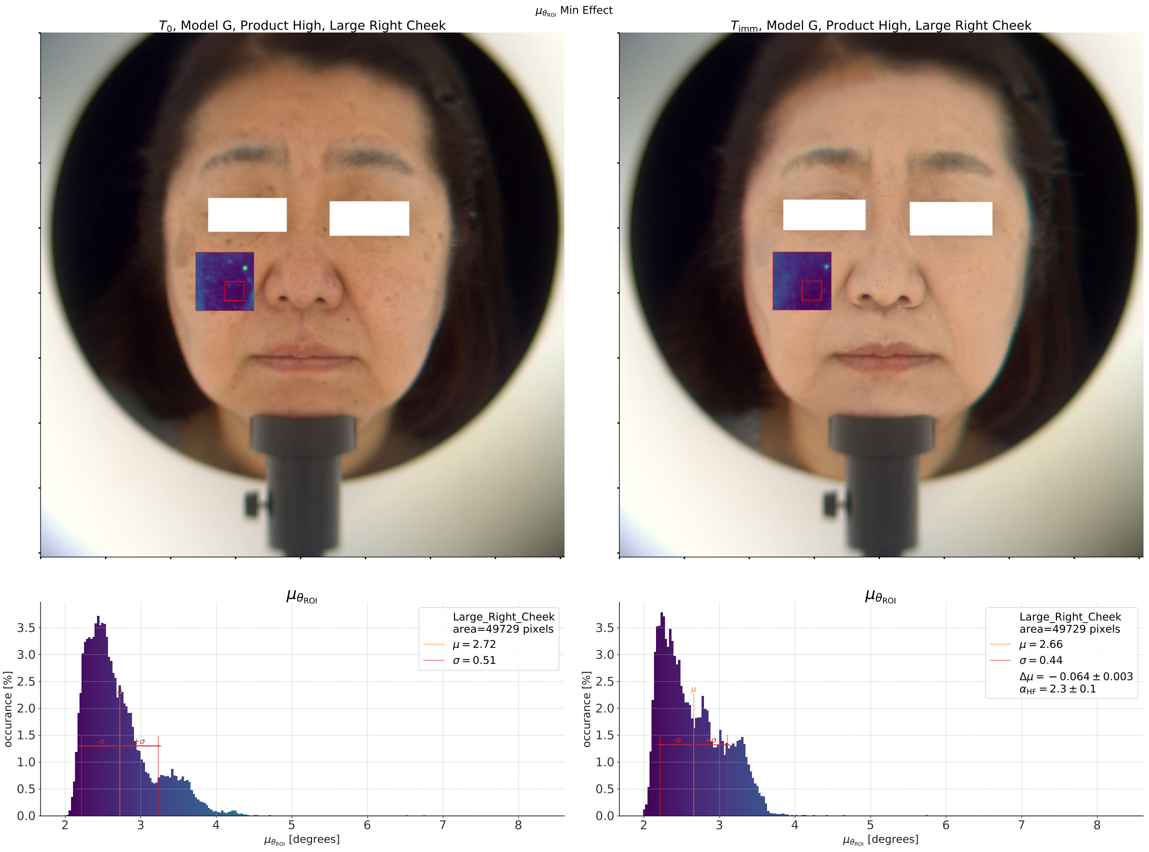

Figure S6. Data Example: Min $\mu_{\theta_{\mathrm{ROI}}}$ result for product *High*.

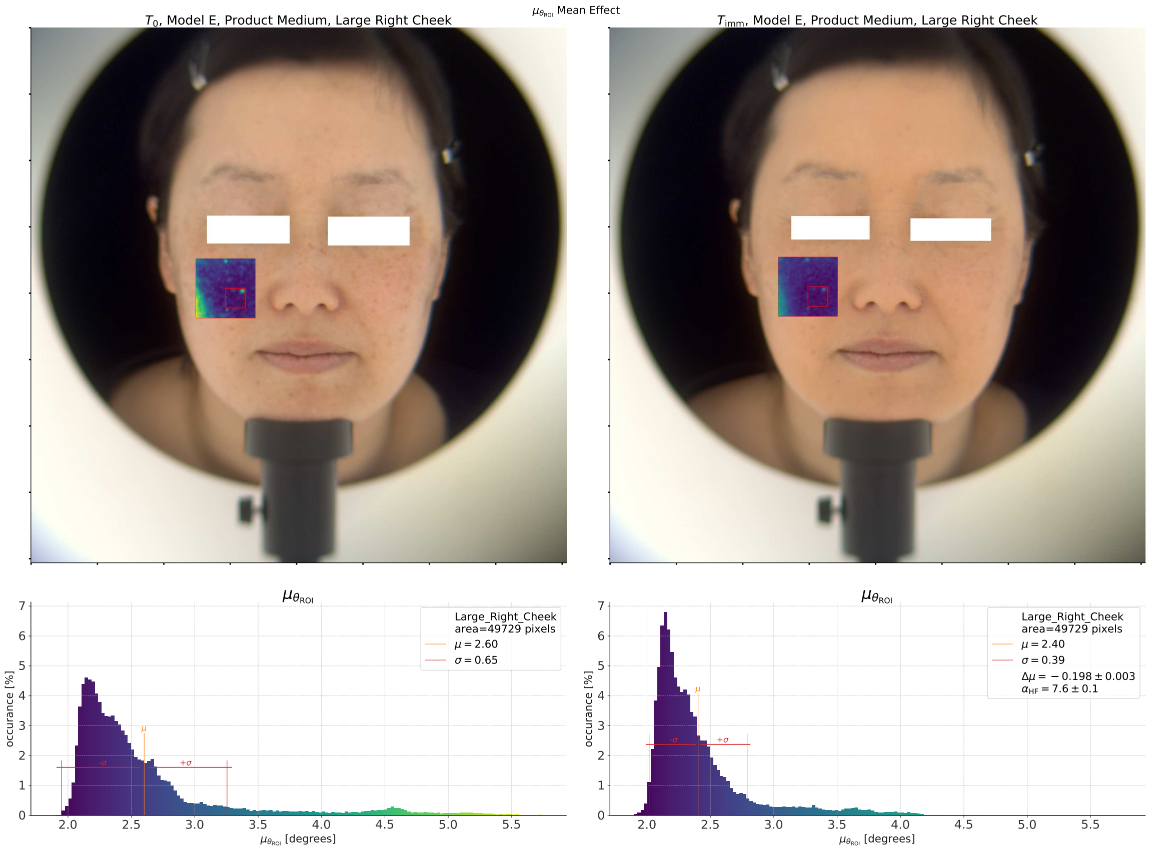

Figure S7. Data Example: Mean $\mu_{\theta_{\mathrm{ROI}}}$ result for product *Medium*.

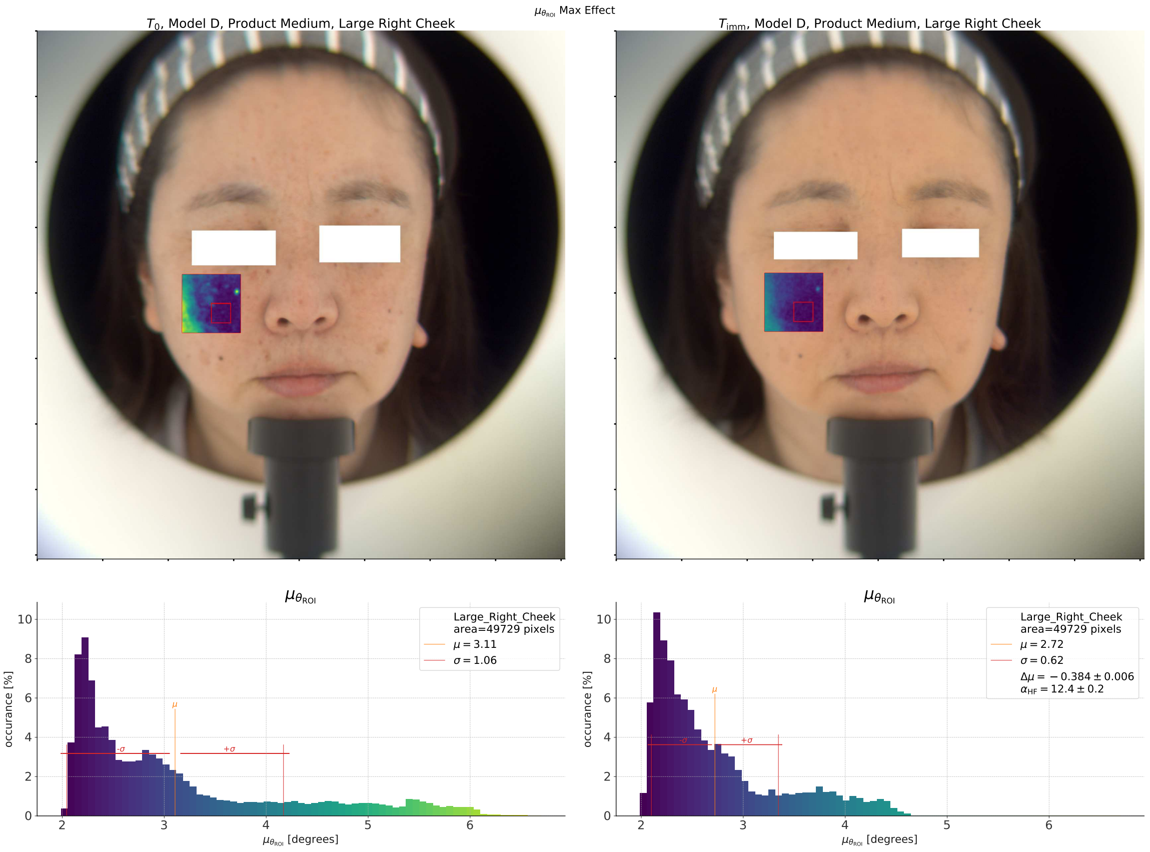

Figure S8. Data Example: Max $\mu_{\theta_{\mathrm{ROI}}}$ result for product *Medium*.

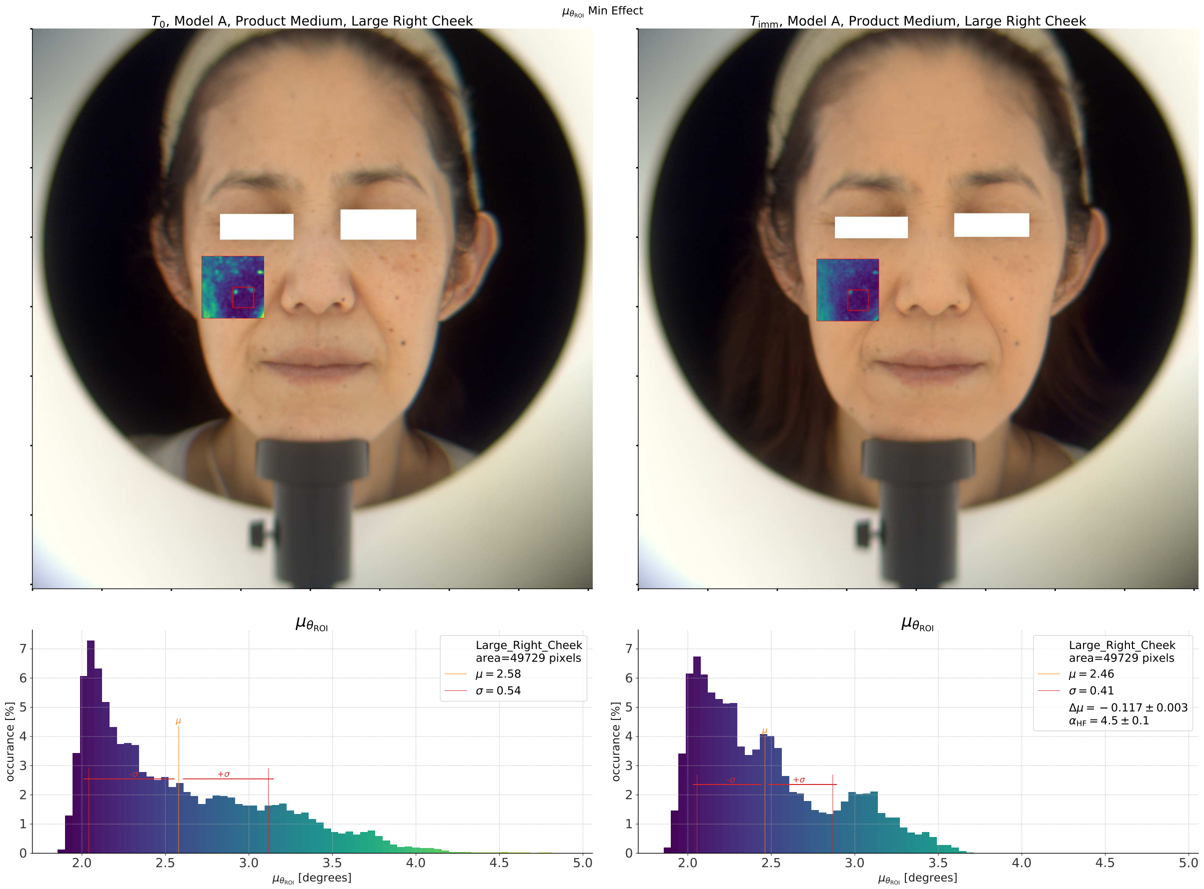

Figure S9. Data Example: Min $\mu_{\theta_{\text{ROI}}}$ result for product *Medium*.
